# The role of extracellular vesicles in phenotypic cancer transformation

**DOI:** 10.2478/raon-2013-0037

**Published:** 2013-07-30

**Authors:** Eva Ogorevc, Veronika Kralj-Iglic, Peter Veranic

**Affiliations:** 1 Laboratory of Biophysics, Faculty of Electrical Engineering, University of Ljubljana, Slovenia; 2 Faculty of Health Sciences, University of Ljubljana, Slovenia; 3 Institute of Cell Biology, Faculty of Medicine, University of Ljubljana, Ljubljana, Slovenia

**Keywords:** extracellular vesicles, exosomes, microvesicles, cancer progression

## Abstract

**Background:**

Cancer has traditionally been considered as a disease resulting from gene mutations. New findings in biology are challenging gene-centered explanations of cancer progression and redirecting them to the non-genetic origins of tumorigenicity. It has become clear that intercellular communication plays a crucial role in cancer progression. Among the most intriguing ways of intercellular communication is that via extracellular vesicles (EVs). EVs are membrane structures released from various types of cells. After separation from the mother membrane, EVs become mobile and may travel from the extracellular space to blood and other body fluids.

**Conclusions:**

Recently it has been shown that tumour cells are particularly prone to vesiculation and that tumour-derived EVs can carry proteins, lipids and nucleic acids causative of cancer progression. The uptake of tumour-derived EVs by noncancerous cells can change their normal phenotype to cancerous. The suppression of vesiculation could slow down tumour growth and the spread of metastases. The purpose of this review is to highlight examples of EV-mediated cancer phenotypic transformation in the light of possible therapeutic applications.

## Introduction

Cancer has been traditionally viewed as a consequence of multistep mutations of genetic material that result in transformation of normal to malignant cells. However, nowadays the mainstream paradigms of cancer development and progression are shifting from strictly genocentric towards epigenetic and other nongenetic interpretations. It is thus relevant to explore the possibility that a normal cell could become malignant without previous genetic mutation. In this article several mechanisms of phenotypic transformation are presented mainly involving transfer of membrane attached receptors for growth factors, RNA molecules or even lipids 1,2,3

It was suggested that intercellular communication plays a crucial role in cancer progression.^1^ Exchange of information is attained through release of specific soluble (or immobilized) signalling molecules and their interaction with corresponding receptors^2^, or through direct cell-to-cell communication that includes gap junctions^3^, cytonems^4^ and tunnelling nanotubes.^5^ In addition to these mechanisms, a highly conserved way of intercellular communication has recently been revealed - communication via extracellular vesicles (EVs).

**FIGURE 1.**
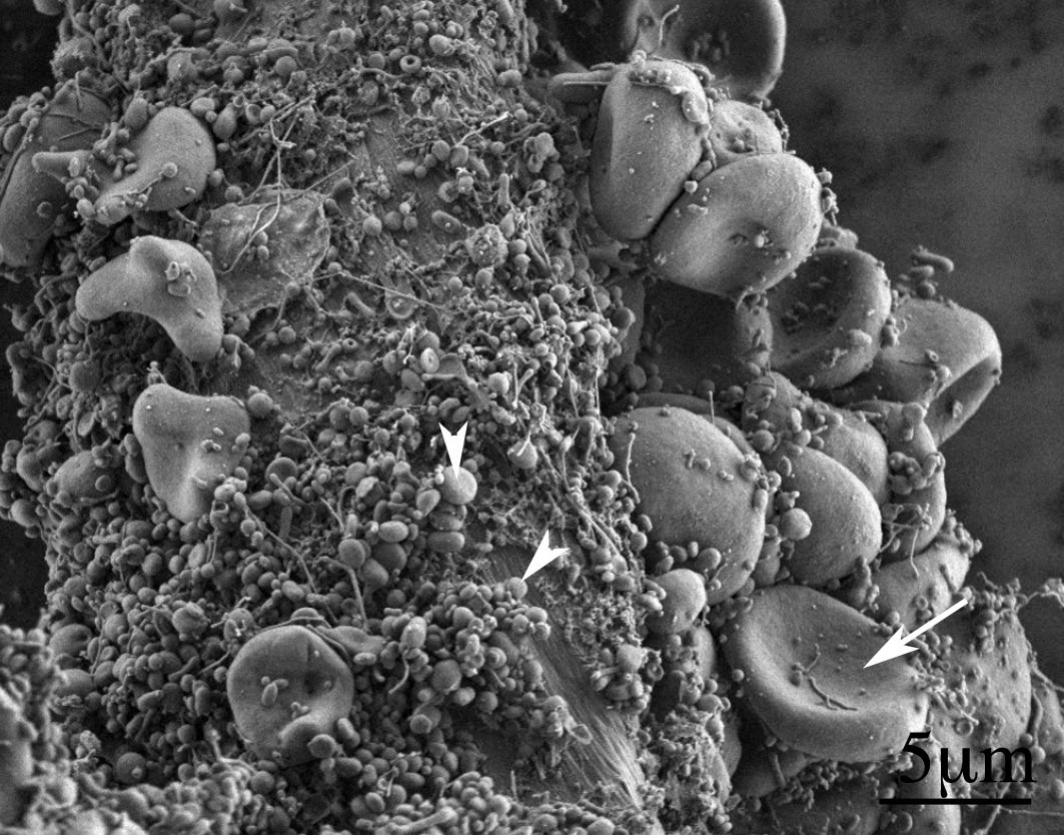
Scanning electron micrograph of an isolate from peripheral blood of a healthy human donor (male, 28 years). A mass of extracellular vesicles (arrowhead) and numerous residual erythrocytes (arrow) can be seen. The image was taken using a Quanta TM 250 FEG scanning electron microscope.

It is considered that EVs are membrane-enclosed compartments, released into the surroundings of practically all cell types, both *in vivo* and *in vitro*.^6^ After separation from the mother membrane, vesicles with various types of cargo become mobile and may travel from the extracellular/intercellular space to blood (Figure 1). Besides in blood-isolates^7^,^8^, EVs were also found in isolates from other body fluids, *i.e.* urine^9^,^10^, ascites^11^,^12^, synovial fluid^8^,^13^, malignant pleural effusions^12^,^14^, bronchial lavage fluid^15^, human semen^16^, breast milk^17^, pregnancy-associated sera^18^, amniotic fluid^19^, ocular fluids^20^ and human saliva.^21^ The vesicles detectable in isolates *in vitro* and *in vivo* represent a mixed population of various sizes and origins. To date no consensus regarding their classification and nomenclature was reached to distinguish between different types of vesicles. In this work we do not consider the apoptotic bodies (usually larger than 1 μm) which are released from the cell in the final stage of programmed cell death.

The content of EVs depends on the cell of origin and the mechanism of vesicle generation. They were found to transfer surface-bound receptors and their ligands, proteins, genetic material, infectious particles, prions and probably even organelles between cells.^22^ A fascinating feature of EVs is that they present multiple epitopes to the recipient cells and therefore on one hand carry signalling molecules for phenotypic transformation and, on the other hand, serve as a cell mechanism to get rid of unwanted constitutents.^23^

**FIGURE 2.**
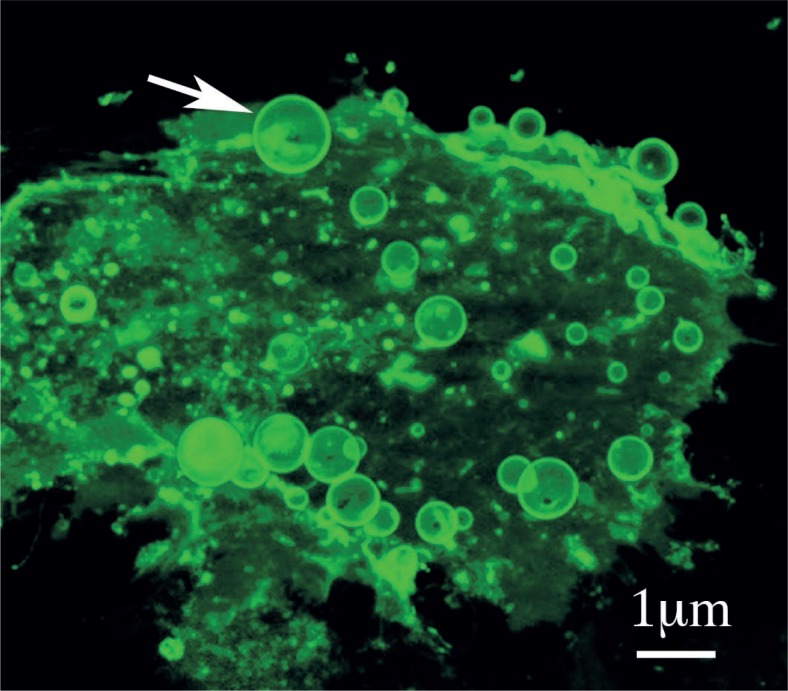
A micrograph presenting multiple vesicles budding from a cell of a urothelial cancer cell line T24 labeled with coleratoxin B - FITC. Arrow points to a budding vesicle. The micrograph is a threedimensional reconstruction of optical sections done by a fluorescence microscope.

Tumour-derived EVs (Figure 2) represent an important component of the tumour microenvironment^22^, but can also take part in altering non-cancerous counterparts (cells) thus facilitating tumour growth and invasion^11^, angiogenesis^24^, metastasis^25^, chemoresistance^26^,^27^, immune evasion^28^,^29^ and escape from cell death.^30^ An increased number of circulating EVs were found in blood isolates of patients with gastrointestinal cancer.^31^–^33^ It is, however, important to bear in mind that the EVs found in blood isolates are not necesarilly the native circulating vesicles but can also be formed during sampling and isolation procedures due to exposure of the cells to thermal and mechanical stress.^6^ Nevertheless, studying EVs isolated from blood and other body fluids of cancer patients is of special interest, not only because cancer cells are particularly prone to vesiculation, but also because of greater vulnerability and fragmentation of blood cells (platelets) in cancer patients, which could be reflected in a higher concentration of EVs in blood-isolates^6^ which could be used as a valuable diagnostic marker.^2^

### Formation of extracellular vesicles

The exact mechanisms underlying the formation of EVs have not yet been fully elucidated, but it seems that vesiculation can be either an extremely well regulated process, or a random, non-specific event associated with, for example, disintegration of the plasma membrane. It is important to realize that general mechanisms of membrane vesiculation can also play a pivotal role in the progression of disease.

**FIGURE 3.**
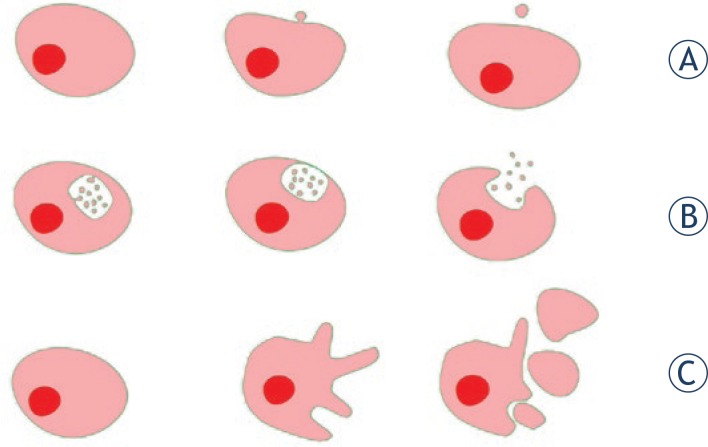
Formation of extracellular vesicles in tumor cells. A. Budding of plasma membrane. B. Release of exosomes after fusion of multivesicular body with plasma membrane. C. Non-apoptotic blebbing.

Membrane vesiculation takes place in the last phase of membrane budding when the bud is pinched off from the membrane to become a free vesicle (Figure 3A). Budding and vesiculation are essential features of the nonspecific biophysical properties of the membrane that impose local and/or global curvature on the membrane in phospholipid bilayer vesicles^34^,^35^, in erythrocytes^36^ and in other cells.^37^,^38^ The packing and distribution of membrane constituents creates local membrane curvature which is consistent with lateral sorting of membrane constituents.^39^ During budding, accumulation of molecules that energetically favour large curvature drives the formation of buds and EVs.^40^ Vesiculation can also be induced by nonlocal events such as an increase or a decrease in the difference between two membrane areas, as described within the bilayer couple concept.^41^–^43^

There is a codependence between membrane shape and structure; moreover, membrane curvature is determined by the shapes of membrane constituents and their interactions.^44^ Sphingolipids, for example, are located mainly in the outer leaflet of the plasma membrane bilayer, while glycerophospholipids such as phosphatidylserine and phosphatidylethanolamine can under normal circumstances be found only in the inner leaflet.^45^,^46^ Cholesterol is believed to occur in similar proportions in both leaflets.^47^ This balance is maintained by several enzymes: scramblase, flippase and translocase.^48^ Disruption of membrane asymmetry and consequent bending of the membrane can occur spontaneously or by an energy-requiring process. Further, the composition and configuration of membrane layer areas are affected by pathophysiological processes such as cell activation, hypoxia, irradiation, oxidative injury, exposure to complement proteins and exposure to shear stress.^22^ Relocation of phosphatidylserine and phosphatidylethanolamine from the inner to the outer leaflet of the plasma membrane is associated with membrane budding and formation of EVs.^49^ EVs are formed in the last stage of the budding process, and thus their surfaces expose large amounts of phosphatidylserine^50^ which can be used for the capture of EVs by phosphatidylserine receptors, such as Annexin V.^51^

Additionally EVs seem to be enriched in proteins and lipids associated with membrane rafts.^50^,^52^ Consistent with this, much experimental and theoretical evidence indicates the importance of membrane rafts in the process of membrane budding and vesiculation.^48^ Membrane rafts are small (10–200 nm) relatively heterogeneous dynamic structures with an increased concentration of cholesterol and sphingolipids.^53^,^54^ Potential roles of membrane rafts in membrane transport were proposed: they may serve as platforms for the inclusion of sorting receptors and cargo molecules, as sites for organizing the membrane cytoskeleton, or as sites for organizing vesicle docking and fusion processes.^55^

Other pathways leading to curvature and subsequent budding of membranes include an increase of intracellular Ca^2+^ inhibiting translocase, activating scramblase and resulting in loss of membrane asymmetry^48^, the reorganization of the cytoskeleton^48^,^56^, and the presence of protein and lipid driving forces since adding a protein or lipid to just one monolayer might cause asymmetry of monolayer areas and thereby increase the intrinsic curvature of the whole bilayer.^57^

Membrane budding can be followed by membrane fission, which is still a subject of some debate, but several ideas supporting the pivotal role of endophilin I and dynamin in this process have been suggested.^58^,^59^

EVs can also be formed in processes distinct from those already mentioned. EVs smaller than 100 nm, usually called exosomes, are formed by exocytosis after the assembly of several endosomes into a multivesicular body, exiting the endosomal pathway and fusion with the plasma membrane (Figure 3B).^50^,^60^,^61^ Peculiarly large EVs (1 –10 mm) can be formed as a result of nonapoptotic blebbing (Figure 3C).^62^ This relatively rapid process of EV-formation is caused by actomyosin contractions near the cortical cytoskeleton. The force required for subsequent bleb retraction is generated by actin filaments.^62^

### Interaction of extracellular vesicles with target cells

It is indicated that EVs interact with the membranes of recipient cells. The precise mechanisms of uptake of EVs are still poorly understood, yet it is becoming increasingly evident that their uptake can induce activation of specific signal transduction cascades and thereby influence the physiological or pathological state of recipient cells.^23^,^63^

Several types of interactions were proposed involving adhesion of vesicle molecules to cellular surface receptors (receptor-mediated uptake), endocytosis (phagocytosis) and fusion with the plasma membrane.^23^,^64^

**FIGURE 4.**
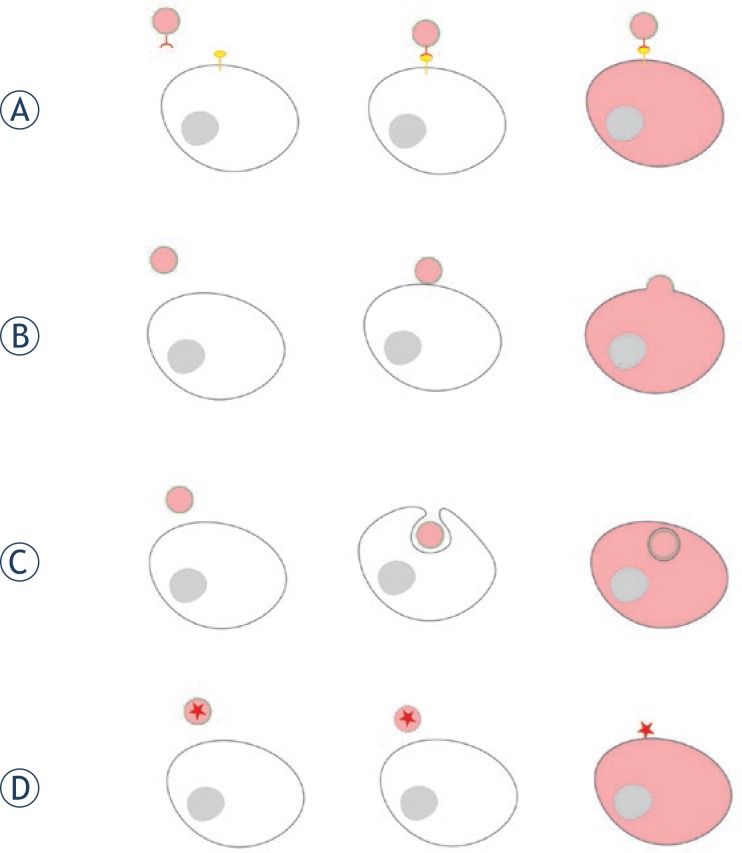
Interaction of extracellular vesicles with recipient cell. (A). Adhesion of vesicle molecules to recipient cell surface receptors. (B). Fusion of vesicle with plasma membrane of a recipient cell. (C). Endocytic / phagocytic uptake. D. Extracellular vesicle breakdown and release of its cargo. Transformed cells with pink cytoplasm.

Potential receptor candidates for interaction with EV-membranes are, notably, receptors for phosphatidylserine. One such receptor is the T-cell immunoglobulin and mucin-domain-containing molecule (TIM) that was described as mediating vesicle uptake.^65^ Segura *et al*.^66^ showed that EVs from mature dendritic cells are enriched in inter-cellular adhesion molecule 1 (ICAM-1), suggesting its role in either helping in capture of EVs by recipient antigen-presenting cells or in favouring T-cell binding of the recipient antigen-presenting cells bearing EVs at their surface (Figure 4A).

The phenomenon of fusion of vesicles with the plasma membrane could be explained by lipid-mediated interactions. Teissier and Pécheur described how lipid rafts, particularly sphingolipids, play a key role in the conformational changes of fusion proteins. These changes lead to interaction of the fusion peptide with the target membrane in viral interactions.^68^ Parolini *et al*. showed that exosomes preferentially fuse with the membranes of tumour cells and that in these interactions the microenvironmental pH acts as a key factor by modulating the lipid compostition of the cell and exosome membranes (Figure 4B).^69^

It seems that phagocytosis is the most effective way of EV-uptake; moreover it has been reported that phagocytic cells have a greater ability for the uptake of EVs than non-phagocytic cells.^67^ Besides phagocytosis clathrin-dependent endocytosis and macropinocytosis were proposed as mechanisms for the uptake of EVs by the ovarian carcinoma cell line (Figure 4C).^70^

Despite all the above discoveries, it is still a question whether the vesicle cargo can be transfered to the recipient cell without the interaction with the membranes. Taraboletti *et al*. showed that acidic pH can induce breakdown of EVs, leading to pericellular release of their cargo and subsequent paracrine activity (Figure 4D).^71^ Furthermore, it has been stated that the breakdown of EVs upon shedding could represent an important signalling mechanism.^72^

### Extracellular vesicles as vehicles in phenotypic malignant transformations

**FIGURE 5.**
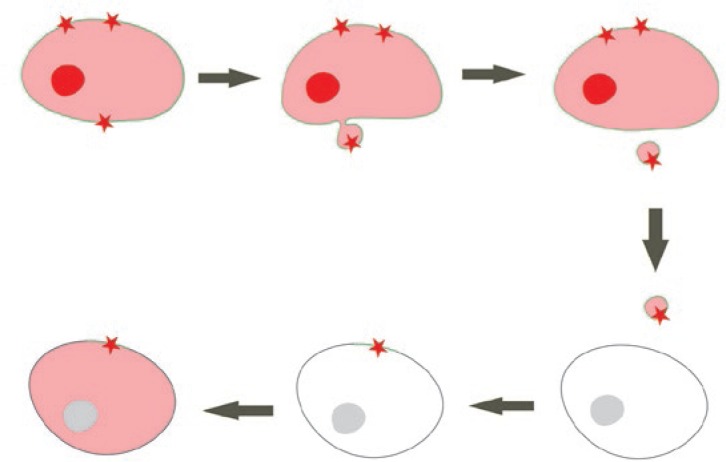
Transfer of oncogenic proteins that induce phenotypic transformation. Tumor cell (pink cytoplasm) with a mutated gene (red nucleus) for membrane protein (EGFRvIII – red asterisk) serve as a donor of this protein which is transferred to a nontumor cell (white cytoplasm). Phenotypic transformation (pink cytoplasm) is induced without mutation (gray nucleus)

When EVs are taken up by recipient cells, they can change the cells’ state, either transitionally or in the long term (Figure 5). Transformation of recipient cells due to EV-transfered cargo was shown to be most efficient if the cell was already to some degree pretransformed or immortalized (stem cells).^73^ It is still unclear whether EVs may be able to exert long-term genomic changes, such as induction of mutations, but it has been brought to light not only that some oncogenes become incorporated into EV-cargo, but also that they can stimulate EV-formation.^74^ Consenquently, EVs can act as vehicles in malignant transformations of normal cells through the transfer of membrane proteins (receptors and receptor-coupled proteins), cytosol proteins, nucleic acids (RNA and DNA) and lipids.^3^

#### Extracellular vesicle-mediated protein transfer

Al Nedawi *et al*. showed that tumour specific growth receptor EGFRvIII can be transferred between glioma cells by EVs, leading to transfer of oncogenic activity, such as activation of transforming signalling pathways (MAPK and Akt), changes in expression of EGFRvIII-regulating genes (VEGF, Bcl-xL, p27), morphological transformation and increase in anchorage-independent growth capacity.^75^

Similar findings were reported in a study by Skog *et al*., where they detect tumour-specific EGFRvIII in serum EVs of glioblastoma patients.^24^ Moreover, they demonstrated that EVs are enriched in angiogenic proteins (interleukin-6, interleukin-8, VEGF) and that they stimulate tubule formation by endothelial cells.^24^

A mechanism that controls metastatic progression through the EV-mediated transfer of another receptor, tyrosine kinase MET, has recently been described. EVs with oncoprotein MET from highly metastatic melanomas increased the metastatic behaviour of primary tumours by permanently educating and mobilizing bone marrow progenitors.^76^

Another example of EV-mediated protein delivery in tumour progession has been described by Sidhu *et al*.^77^ The authors showed that extracellular matrix metalloproteinase inducer (EMMPRIN or CD147) is released from the surface of lung carcinoma cells via EVs which rapidly break down to release bioactive EMMPRIN, that stimulates matrix metalloproteinase expression in fibroblasts, thereby facilitating tumour invasion and metastasis.^77^

Many other proteins have been identified in EVs shed from cancer cells, including among others vascular endothelial growth factor (VEGF)^71^, tetraspanins^64^, heat-shock protein 90α^78^, Mart-1/MelanA, carcinoembryogenic antigen^79^ and HER2.^79^,^80^

#### Extracellular vesicle-mediated horizontal transfer of (epi)genetic information

Recently it has come to light that messenger RNA (mRNA) and various forms of non-coding RNA, such as microRNA (miRNA), act as key players in information transfer between cells.^81^ miRNAs are small noncoding RNA gene products believed to negatively regulate other genes’ expression. Furthermore, there is evidence that miRNA species might act as tumour suppressors and oncogenes.^82^,^83^ As RNA molecules are unstable in plasma or blood^84^,^85^, they should be in some way protected from degradation during systemic transport. Membrane vesicles appear to be ideal candidates for this kind of protection. In fact, it seems that almost all systemically transfered RNA is stored compactly within EVs and is thereby protected from external RNAse.^24^,^81^ Additionally, more permanent modulation of recipient cells may be achieved through uptake of EVs containing nucleic acids. Interestingly, a microarray comparison of mRNA populations in EVs and their donor cells showed that specific mRNA species were detected exclusively in EVs, suggesting a specific packaging mechanism that encapsulates these mRNAs into EVs.^24^,^86^ Several groups have described the key role of EV-mediated mRNA transfer in tumour progression in various types of cancer, such as colorectal adenocarcinoma^87^,^88^, pancreatic adeno-carcinoma^88^, lung carcinoma^88^ and glioblastoma.^24^ The presence of specific miRNA species has been reported in EVs derived either from carcinoma cell-lines or from serum of cancer patients. A study showed that hepatocellular carcinoma cell-derived EVs mediate miRNA transfer and thereby enhance recipient cell growth.^89^ Ohshima *et al*. reported that metastatic gastric cancer cell line releases EVs enriched in let-7-miRNAs, known to negatively regulate Ras genes, leading to maintenance of their oncogenesis.^90^ Another study showed that EVs from the serum of ovarian cancer patients contain specific miRNA signatures and suggested that circulating EVs could potentially be used as surrogate diagnostic markers.^91^

EVs have been found to transfer DNA between cells, but it is important to keep in mind that EV fractions can also consist of apoptotic bodies, known to contain DNA fragments, possibly contributing to genetic changes and tumour progression.^92^ A group recently showed that brain tumour cells release EVs that contain single stranded DNA (ssDNA), including both cDNA and genomic DNA.^93^ The transported DNA contained amplified sequences of the c-Myc oncogene that could be available for horizonzal gene transfer and malignant transformation.^93^

Mitochondrial dysfunction and especially dys-functions caused by mutations of mtDNA have been implicated with a wide range of age-related pathologies, including cancer.^94^ It was reported that EVs from glioblastoma and astrocyte cells contain mitochondrial DNA which can be transferred between cells.^95^

A large part of the mammalian genome is derived from ancient transposable elements, such as DNA-transposons and retrotransposons. While DNA-transposons amplify without any RNA intermediate, retrotransposons need reverse transcriptase to retrotranscribe them before integration into the genome.^96^ The expression of retrotransposons is increased in tumour cells through hypomethylation of the genome^97^; further it has been reported that retrotransposon insertion into the genome triggers mutations in tumorigenesis.^98^ Balaj *et al*. incubated EVs derived from human medulloblastoma cells and enriched in retrotransposon RNAs, especially HERV-K, with HUVEC cells.^93^ After incubation the content of HERV-K in the HUVEC cells was increased up to 60-fold, suggesting the active role of EVs in transfering retrotrasposon sequences to normal surrounding cells.^93^

#### Extracellular vesicle-mediated lipid delivery

Sphingomyelin is a major membrane phospholipid, mostly localized in the outer leaflet of the mammalian plasma membrane.^99^ A significantly increased level of sphingomyelin in the highly metastatic adenocarcinoma cell line was reported in comparison to the lower metastatic variant of adenocarcinoma, suggesting the role of sphingomyelin not only as an important membrane component, but also as a key player in tumour metastasis.^100^

Kim *et al*. showed the importance of sphingomyelin transfer in cancer progression.^101^ Namely, they indicated that sphingomyelin is a major active component in angiogenesis. They also found an increased amount of sphingomyelin in EVs derived from tumour cells compared to that from the plasma membrane.^101^

### Suppression of oncogenic transformation by extracellular vesicles

It has been shown that heparin, usually used for the treatment of thromboembolisms^102^, also has a beneficial effect in suppressing tumour progression in some types of cancer.^103^,^104^ Interestingly, both effects of heparin can be explained by suppression of EV formation on the basis of non-specific biophysical mechanisms. The study, performed on artificial membrane models with controlled lipid composition – giant unilamellar vesicles (GUVs) - showed that budding and vesiculation of membranes can be affected by the surrounding solution.^105^ Theoretically and experimentaly it was shown that molecules and ions in the solution can mediate attractive interactions between membranes and cause adhesion.^106^,^107^ The composition and physical properties of the solution in the vicinity of the membrane^108^–^110^ importantly affect these interactions and it was revealed that in the budding process the bud can adhere back to the mother membrane if the mediating effect of the solution is strong enough.^106^ It was found that plasma contains molecules that mediate attractive interaction between membranes and that added heparin enhances this effect.^105^ A mediating effect was also found for anticoagulant β2-glycoprotein I.^107^,^111^ It was suggested that similar mechanisms may take place in cells, but it is important to note that cell membranes are of more complex composition, making the described mechanisms somewhat distinct.^105^ Nevertheless, substances which mediate attractive interaction between membranes (*e.g*. heparin) are suppressors of membrane vesiculation and can therefore have anticoagulant, antimetastatic and anti-inflammatory effect.^105^

## Conclusion

Recent investigations revealed that invasive tumours can be spread in the body not only by metastases travelling along tissues or being transported by body fluids and so seeding new tumours after anchoring to targeted tissues. Tumours can also be spread by much smaller carriers in the form of EVs containing genetic information or mutant growth factor receptors that are permanently active and provoke over-inducing signalling of cell division. Transfer of such vesicles can occur over short distances to neighbouring cells or long distances by body fluids. By finding appropriate target cells the transferred transforming molecules can induce cell transformation and cause cancer progression most efficiently in already immortalized precancerous or stem cells. As tumor cell transformation is usually a multistep process including several consecutive mutations it can be concluded that the transfer of a transforming molecules can serve as one of the steps in this process. By carrying certain enzymes such as metalloproteinases, the EVs can adapt the microenvironment of tumour cells in favour of metastatic dissemination or implantation into certain tissues. Blocking the spreading of EVs, by the use of molecules attaching the vesicles to the vesiculating cells could possibly slow down tumour growth or the spread of metastases. On the other hand, screening of cancer genetic markers transported by EVs could improve diagnostic methods for detection of certain cancerous diseases. A thorough understanding of the biological mechanisms involved in intercellular communication by EVs could provide a key complement to genetic factors in determination of cancer progression, while their controlled manipulation will likely develop into a powerful weapon in the battlefield of oncology.

## References

[1] Al-Nedawi K, Meehan B, Rak J (2009). Microvesicles: messengers and mediators of tumor progression. Cell Cycle.

[2] Rak J (2010). Microparticles in cancer. Semin Thromb Hemost.

[3] Pap E (2011). The role of microvesicles in malignancies. Adv Exp Med Biol.

[4] Camussi G, Deregibus MC, Bruno S, Grange C, Fonsato V, Tetta C (2011). Exosome/microvesicle-mediated epigenetic reprogramming of cells. Am J Cancer Res.

[5] Veranic P, Lokar M, Schutz GJ, Weghuber J, Wieser S, Hagerstrand H (2008). Different types of cell-to-cell connections mediated by nanotubular structures. Biophys J.

[6] Sustar V, Bedina-Zavec A, Stukelj R, Frank M, Bobojevic G, Jansa R (2011). Nanoparticles isolated from blood: a reflection of vesiculability of blood cells during the isolation process. Int J Nanomedicine.

[7] Wolf P (1967). The nature and significance of platelet products in human plasma. Br J Haematol.

[8] Junkar I, Sustar V, Frank M, Jansa V, Bedina Zavec A, Rozman B (2009). Blood and synovial microparticles as revealed by atomic force and scanning electron microscope. Open Autoimmun J.

[9] Pisitkun T, Shen RF, Knepper MA (2004). Identification and proteomic profiling of exosomes in human urine. Proc Natl Acad Sci USA.

[10] Gonzales P, Pisitkun T, Knepper MA (2008). Urinary exosomes: is there a future?. Nephrol Dial Transplant.

[11] Graves LE, Ariztia EV, Navari JR, Matzel HJ, Stack MS, Fishman DA (2004). Proinvasive properties of ovarian cancer ascites-derived membrane vesicles. Cancer Res.

[12] Mrvar-Brecko A, Sustar V, Jansa V, Stukelj R, Jansa R, Mujagic E (2010). Isolated microvesicles from peripheral blood and body fluids as observed by scanning electron microscope. Blood Cells Mol Dis.

[13] Skriner K, Adolph K, Jungblut PR, Burmester GR (2006). Association of citrullinated proteins with synovial exosomes. Arthritis Rheum.

[14] Bard MP, Hegmans JP, Hemmes A, Luider TM, Willemsen R, Severijnen LA (2004). Proteomic analysis of exosomes isolated from human malignant pleural effusions. Am J Respir Cell Mol Biol.

[15] Admyre C, Grunewald J, Thyberg J, Gripenback S, Tornling G, Eklund A (2003). Exosomes with major histocompatibility complex class II and costimulatory molecules are present in human BAL fluid. Eur Respir J.

[16] Sullivan R, Saez F, Girouard J, Frenette G (2005). Role of exosomes in sperm maturation during the transit along the male reproductive tract. Blood Cells Mol Dis.

[17] Admyre C, Johansson SM, Qazi KR, Filen JJ, Lahesmaa R, Norman M (2007). Exosomes with immune modulatory features are present in human breast milk. J Immunol.

[18] Taylor DD, Akyol S, Gercel-Taylor C (2006). Pregnancy-associated exosomes and their modulation of T cell signaling. J Immunol.

[19] Asea A, Jean-Pierre C, Kaur P, Rao P, Linhares IM, Skupski D (2008). Heat shock protein-containing exosomes in mid-trimester amniotic fluids. J Reprod Immunol.

[20] Perkumas KM, Hoffman EA, McKay BS, Allingham RR, Stamer WD (2007). Myocilin-associated exosomes in human ocular samples. Exp Eye Res.

[21] Ogawa Y, Kanai-Azuma M, Akimoto Y, Kawakami H, Yanoshita R (2008). Exosome-like vesicles with dipeptidyl peptidase IV in human saliva. Biol Pharm Bull.

[22] Ratajczak J, Wysoczynski M, Hayek F, Janowska-Wieczorek A, Ratajczak MZ (2006). Membrane-derived microvesicles: important and underappreciated mediators of cell-to-cell communication. Leukemia.

[23] van der Vos KE, Balaj L, Skog J, Breakefield XO (2011). Brain Tumor Microvesicles: Insights into Intercellular Communication in the Nervous System. Cell Mol Neurobiol.

[24] Skog J, Wurdinger T, van Rijn S, Meijer DH, Gainche L, Sena-Esteves M (2008). Glioblastoma microvesicles transport RNA and proteins that promote tumour growth and provide diagnostic biomarkers. Nat Cell Biol.

[25] Janowska-Wieczorek A, Wysoczynski M, Kijowski J, Marquez-Curtis L, Machalinski B, Ratajczak J (2005). Microvesicles derived from activated platelets induce metastasis and angiogenesis in lung cancer. Int J Cancer.

[26] Safaei R, Larson BJ, Cheng TC, Gibson MA, Otani S, Naerdemann W (2005). Abnormal lysosomal trafficking and enhanced exosomal export of cisplatin in drug-resistant human ovarian carcinoma cells. Mol Cancer Ther.

[27] Shedden K, Xie XT, Chandaroy P, Chang YT, Rosania GR (2003). Expulsion of small molecules in vesicles shed by cancer cells: association with gene expression and chemosensitivity profiles. Cancer Res.

[28] Hakulinen J, Junnikkala S, Sorsa T, Meri S (2004). Complement inhibitor membrane cofactor protein (MCP; CD46) is constitutively shed from cancer cell membranes in vesicles and converted by a metalloproteinase to a functionally active soluble form. Eur J Immunol.

[29] Valenti R, Huber V, Filipazzi P, Pilla L, Sovena G, Villa A (2006). Human tumor-released microvesicles promote the differentiation of myeloid cells with transforming growth factor-beta-mediated suppressive activity on T lymphocytes. Cancer Res.

[30] Abid Hussein MN, Boing AN, Sturk A, Hau CM, Nieuwland R (2007). Inhibition of microparticle release triggers endothelial cell apoptosis and detachment. Thromb Haemost.

[31] Kim HK, Song KS, Park YS, Kang YH, Lee YJ, Lee KR (2003). Elevated levels of circulating platelet microparticles, VEGF, IL-6 and RANTES in patients with gastric cancer: possible role of a metastasis predictor. Eur J Cancer.

[32] Jansa R, Sustar V, Frank M, Susanj P, Bester J, Mancek-Keber M (2008). Number of microvesicles in peripheral blood and ability of plasma to induce adhesion between phospholipid membranes in 19 patients with gastrointestinal diseases. Blood Cells Mol Dis.

[33] Baran J, Baj-Krzyworzeka M, Weglarczyk K, Szatanek R, Zembala M, Barbasz J (2010). Circulating tumour-derived microvesicles in plasma of gastric cancer patients. Cancer Immunol Immunother.

[34] Lipowsky R (1991). The conformation of membranes. Nature.

[35] Kralj-Iglic V, Babnik B, Gauger DR, May S, Iglic A (2006). Quadrupolar ordering of phospholipid molecules in narrow necks of phospholipid vesicles. J Stat Phys.

[36] Hagerstrand H, Isomaa B (1992). Morphological characterization of exovesicles and endovesicles released from human erythrocytes following treatment with amphiphiles. Biochim Biophys Acta.

[37] Black PH (1980). Shedding from Normal and Cancer-Cell Surfaces. New Engl J Med.

[38] Kralj-Iglic V, Batista U, Hägerstrand H, Iglic A, Majhenc J, Sok M (1998). On mechanisms of cell plasma membrane vesiculation. Radiol Oncol.

[39] Kralj-Iglic V, Veranic P, Leitmannova Liu A (2007). Curvature-Induced Sorting of Bilayer Membrane Constituents and Formation of Membrane Rafts. Advances in planar lipid bilayers and liposomes.

[40] Kralj-Iglic V, Iglic A, Hagerstrand H, Peterlin P (2000). Stable tubular microexovesicles of the erythrocyte membrane induced by dimeric amphiphiles. Phys Rev E Stat Phys Plasmas Fluids Relat Interdiscip Topics.

[41] Sheetz MP, Singer SJ (1974). Biological-membranes as bilayer couples - molecular mechanism of drug-erythrocyte interactions. Proc Natl Acad Sci USA.

[42] Helfrich W (1974). Blocked lipid exchange in bilayers and its possible influence on the shape of vesicles. Z. Naturforsch.

[43] Evans EA (1974). Bending resistance and chemically induced moments in membrane bilayers. Biophys J.

[44] Kralj-Iglic V (2012). Stability of membranous nanostructures: a possible key mechanism in cancer progression. Int J Nanomedicine.

[45] Zachowski A, Devaux PF (1990). Transmembrane movements of lipids. Experientia.

[46] Sims PJ, Wiedmer T (2001). Unraveling the mysteries of phospholipid scrambling. Thromb Haemost.

[47] Wydro P, Hac-Wydro K (2007). Thermodynamic description of the interactions between lipids in ternary Langmuir monolayers: the study of cholesterol distribution in membranes. J Phys Chem B.

[48] Pap E, Pallinger E, Pasztoi M, Falus A (2009). Highlights of a new type of intercellular communication: microvesicle-based information transfer. Inflamm Res.

[49] van Meer G (2011). Dynamic transbilayer lipid asymmetry. Csh Perspect Biol.

[50] Camussi G, Deregibus MC, Bruno S, Cantaluppi V, Biancone L (2010). Exosomes/ microvesicles as a mechanism of cell-to-cell communication. Kidney Int.

[51] Davizon P, Lopez JA (2009). Microparticles and thrombotic disease. Curr Opin Hematol.

[52] Mrówczyńska L, Salzer U, Iglič A, Hägerstrand H (2011). Curvature factor and membrane solubilisation, with particular reference to membrane rafts. Cell Biol Int.

[53] Simons K, Ikonen E (1997). Functional rafts in cell membranes. Nature.

[54] Brown DA, London E (1998). Functions of lipid rafts in biological membranes. Annu Rev Cell Dev Bi.

[55] Ikonen E (2001). Roles of lipid rafts in membrane transport. Curr Opin Cell Biol.

[56] Flaumenhaft R (2006). Formation and fate of platelet microparticles. Blood Cells Mol Dis.

[57] Huttner WB, Zimmerberg J (2001). Implications of lipid microdomains for membrane curvature, budding and fission. Curr Opin Cell Biol.

[58] Schmidt A, Wolde M, Thiele C, Fest W, Kratzin H, Podtelejnikov AV (1999). Endophilin I mediates synaptic vesicle formation by transfer of arachidonate to lysophosphatidic acid. Nature.

[59] Kozlov MM (2001). Fission of biological membranes: interplay between dynamin and lipids. Traffic.

[60] Heijnen HFG, Schiel AE, Fijnheer R, Geuze HJ, Sixma JJ (1999). Activated platelets release two types of membrane vesicles: Microvesicles by surface shedding and exosomes derived from exocytosis of multivesicular bodies and alpha-granules. Blood.

[61] Pap E, Pallinger E, Falus A (2011). The role of membrane vesicles in tumorigenesis. Crit Rev Oncol Hematol.

[62] Di Vizio D, Kim J, Hager MH, Morello M, Yang W, Lafargue CJ (2009). Oncosome formation in prostate cancer: association with a region of frequent chromosomal deletion in metastatic disease. Cancer Res.

[63] Del Conde I, Shrimpton CN, Thiagarajan P, Lopez JA (2005). Tissue-factor-bearing microvesicles arise from lipid rafts and fuse with activated platelets to initiate coagulation. Blood.

[64] Kharaziha P, Ceder S, Li Q, Panaretakis T (2012). Tumor cell-derived exosomes: A message in a bottle. Biochim Biophys Acta.

[65] Miyanishi M, Tada K, Koike M, Uchiyama Y, Kitamura T, Nagata S (2007). Identification of Tim4 as a phosphatidylserine receptor. Nature.

[66] Segura E, Nicco C, Lombard B, Veron P, Raposo G, Batteux F (2005). ICAM-1 on exosomes from mature dendritic cells is critical for efficient naive T-cell priming. Blood.

[67] Feng D, Zhao WL, Ye YY, Bai XC, Liu RQ, Chang LF (2010). Cellular Internalization of exosomes occurs through phagocytosis. Traffic.

[68] Teissier E, Pecheur EI (2007). Lipids as modulators of membrane fusion mediated by viral fusion proteins. Eur Biophys J.

[69] Parolini I, Federici C, Raggi C, Lugini L, Palleschi S, De Milito A (2009). Microenvironmental pH is a key factor for exosome traffic in tumor cells. J Biol Chem.

[70] Escrevente C, Keller S, Altevogt P, Costa J (2011). Interaction and uptake of exosomes by ovarian cancer cells. BMC Cancer.

[71] Taraboletti G, D’Ascenzo S, Giusti I, Marchetti D, Borsotti P, Millimaggi D (2006). Bioavailability of VEGF in tumor-shed vesicles depends on vesicle burst induced by acidic pH. Neoplasia.

[72] Cocucci E, Racchetti G, Meldolesi J (2009). Shedding microvesicles: artefacts no more. Trends Cell Biol.

[73] Rak J, Guha A (2012). Extracellular vesicles - vehicles that spread cancer genes. Bioessays.

[74] Lee TH, D’Asti E, Magnus N, Al-Nedawi K, Meehan B, Rak J (2011). Microvesicles as mediators of intercellular communication in cancer - the emerging science of cellular ‘debris’. Semin Immunopathol.

[75] Al-Nedawi K, Meehan B, Micallef J, Lhotak V, May L, Guha A (2008). Intercellular transfer of the oncogenic receptor EGFRvIII by microvesicles derived from tumour cells. Nat Cell Biol.

[76] Peinado H, Aleckovic M, Lavotshkin S, Matei I, Costa-Silva B, Moreno-Bueno G (2012). Melanoma exosomes educate bone marrow progenitor cells toward a pro-metastatic phenotype through MET. Nat Med.

[77] Sidhu SS, Mengistab AT, Tauscher AN, LaVail J, Basbaum C (2004). The microvesicle as a vehicle for EMMPRIN in tumor-stromal interactions. Oncogene.

[78] McCready J, Sims JD, Chan D, Jay DG (2010). Secretion of extracellular hsp90alpha via exosomes increases cancer cell motility: a role for plasminogen activation. BMC Cancer.

[79] Andre F, Schartz NE, Movassagh M, Flament C, Pautier P, Morice P (2002). Malignant effusions and immunogenic tumour-derived exosomes. Lancet.

[80] Koga K, Matsumoto K, Akiyoshi T, Kubo M, Yamanaka N, Tasaki A (2005). Purification, characterization and biological significance of tumor-derived exosomes. Anticancer Res.

[81] Dinger ME, Mercer TR, Mattick JS (2008). RNAs as extracellular signaling molecules. J Mol Endocrinol.

[82] Lagos-Quintana M, Rauhut R, Lendeckel W, Tuschl T (2001). Identification of novel genes coding for small expressed RNAs. Science.

[83] Esquela-Kerscher A, Slack FJ (2006). Oncomirs - microRNAs with a role in cancer. Nat Rev Cancer.

[84] Tsui NB, Ng EK, Lo YM (2002). Stability of endogenous and added RNA in blood specimens, serum, and plasma. Clin Chem.

[85] Tsui NB, Ng EK, Lo YM (2006). Molecular analysis of circulating RNA in plasma. Methods Mol Biol.

[86] Valadi H, Ekstrom K, Bossios A, Sjostrand M, Lee JJ, Lotvall JO (2007). Exosome-mediated transfer of mRNAs and microRNAs is a novel mechanism of genetic exchange between cells. Nat Cell Biol.

[87] Hong BS, Cho JH, Kim H, Choi EJ, Rho S, Kim J (2009). Colorectal cancer cell-derived microvesicles are enriched in cell cycle-related mRNAs that promote proliferation of endothelial cells. BMC Genomics.

[88] Baj-Krzyworzeka M, Szatanek R, Weglarczyk K, Baran J, Urbanowicz B, Branski P (2006). Tumour-derived microvesicles carry several surface determinants and mRNA of tumour cells and transfer some of these determinants to monocytes. Cancer Immunol Immunother.

[89] Kogure T, Lin WL, Yan IK, Braconi C, Patel T (2011). Intercellular nanovesicle-mediated microRNA transfer: a mechanism of environmental modulation of hepatocellular cancer cell growth. Hepatology.

[90] Ohshima K, Inoue K, Fujiwara A, Hatakeyama K, Kanto K, Watanabe Y (2010). Let-7 microRNA family is selectively secreted into the extracellular environment via exosomes in a metastatic gastric cancer cell line. PLoS One.

[91] Taylor DD, Gercel-Taylor C (2008). MicroRNA signatures of tumor-derived exosomes as diagnostic biomarkers of ovarian cancer. Gynecol Oncol.

[92] Bergsmedh A, Szeles A, Henriksson M, Bratt A, Folkman MJ, Spetz AL (2001). Horizontal transfer of oncogenes by uptake of apoptotic bodies. Proc Natl Acad Sci USA.

[93] Balaj L, Lessard R, Dai L, Cho YJ, Pomeroy SL, Breakefield XO (2011). Tumour microvesicles contain retrotransposon elements and amplified oncogene sequences. Nat Commun.

[94] Desler C, Marcker ML, Singh KK, Rasmussen LJ (2011). The importance of mitochondrial DNA in aging and cancer. J Aging Res.

[95] Guescini M, Genedani S, Stocchi V, Agnati LF (2010). Astrocytes and Glioblastoma cells release exosomes carrying mtDNA. J Neural Transm.

[96] Bannert N, Kurth R (2004). Retroelements and the human genome: new perspectives on an old relation. Proc Natl Acad Sci USA.

[97] Cordaux R, Batzer MA (2009). The impact of retrotransposons on human genome evolution. Nat Rev Genet.

[98] Wiemels JL, Hofmann J, Kang M, Selzer R, Green R, Zhou M (2008). Chromosome 12p deletions in TEL-AML1 childhood acute lymphoblastic leukemia are associated with retrotransposon elements and occur postnatally. Cancer Res.

[99] Bretscher MS (1973). Membrane structure: some general principles. Science.

[100] Dahiya R, Boyle B, Goldberg BC, Yoon WH, Konety B, Chen K (1992). Metastasis-associated alterations in phospholipids and fatty acids of human prostatic adenocarcinoma cell lines. Biochem Cell Biol.

[101] Kim CW, Lee HM, Lee TH, Kang C, Kleinman HK, Gho YS (2002). Extracellular membrane vesicles from tumor cells promote angiogenesis via sphingomyelin. Cancer Res.

[102] McGarry LJ, Thompson D (2004). Retrospective database analysis of the prevention of venous thromboembolism with low-molecular-weight heparin in acutely III medical inpatients in community practice. Clin Ther.

[103] Smorenburg SM, Hettiarachchi RJ, Vink R, Buller HR (1999). The effects of unfractionated heparin on survival in patients with malignancy-a systematic review. Thromb Haemost.

[104] Stevenson JL, Choi SH, Wahrenbrock M, Varki A, Varki NM (2005). Heparin effects in metastasis and Trouseeau syndrome: anticoagulation is not the primary mechanism. Haem Rep.

[105] Sustar V, Jansa R, Frank M, Hagerstrand H, Krzan M, Iglic A (2009). Suppression of membrane microvesiculation - a possible anticoagulant and anti-tumor progression effect of heparin. Blood Cells Mol Dis.

[106] Urbanija J, Tomsic N, Lokar M, Ambrozic A, Cucnik S, Rozman B (2007). Coalescence of phospholipid membranes as a possible origin of anticoagulant effect of serum proteins. Chem Phys Lipids.

[107] Urbanija J, Babnik B, Frank M, Tomsic N, Rozman B, Kralj-Iglic V (2008). Attachment of beta 2-glycoprotein I to negatively charged liposomes may prevent the release of daughter vesicles from the parent membrane. Eur Biophys J.

[108] May S, Iglič A, Reščič J, Maset S, Bohinc K (2008). Bridging like-charged macroions through long divalent rod-like ions. J Phys Chem B.

[109] Velikonja A, Perutkova Š, Gongadze E, Kramar P, Polak A, Maček-Lebar A, Iglič A (2013). Monovalent ions and water dipoles in contact with dipolar zwitterionic lipid headroups - theory and MD simulations. Int J Mol Sci.

[110] Gongadze E, Iglič A (2013). Excluded volume effect of counterions and water dipoles near a highly charged surface due to a rotationally averaged Boltzmann factor for water dipoles. Gen Phys Biophys.

[111] Ambrožič A, Čučnik S, Tomšič N, Urbanija J, Lokar M, Babnik B (2006). Interaction of giant phospholipid vesicles containing cardiolipin and cholesterol with beta 2-glycoprotein-I and anti-beta2-glycoprotein-I antibodies. Autoimmun Rev.

